# Commentary: Effectiveness of theta burst vs. high-frequency repetitive transcranial magnetic stimulation in patients with depression (THREE-D): a randomized non-inferiority trial

**DOI:** 10.3389/fnhum.2018.00255

**Published:** 2018-06-25

**Authors:** Cuilan Han, Zhongming Chen, Lin Liu

**Affiliations:** Department of Psychiatry, Ningbo Kangning Hospital, Ningbo, China

**Keywords:** brain stimulation, depression, treatment, iTBS, TMS, tDCS

Transcranial magnetic stimulation (TMS) has been widely adopted for clinical treatments for depression and many other psychiatric disorders (Brunoni et al., 2017). Previous clinical trials demonstrated numerous benefits of TMS therapy over pharmacological treatments, in terms of minor side effects, well tolerated for most patients, and the comparable effectiveness (Fitzgerald and Daskalakis, 2012). However, in clinical practices, a half-hour TMS therapy could be slow and cost weeks to be fully effective; the patients need to 5 days per week during the periods, which is time consuming and much less convenient than taking pills. Should a faster and more concentrated TMS protocol be effective for depression or other psychiatric disorders, it might reduce the treatment time length during the whole treatment period (Fitzgerald et al., 2018). A recent study by Blumberger et al. demonstrated the possibility to perform theta burst stimulation protocol on depression patients and achieve similar antidepressant effects than classical high frequency ones (Blumberger et al., 2018).

The pulse concentration of TMS protocol determines the amount of neural excitation generated in the targeted cortex (Walsh and Cowey, 2000). It is conceivable that some non-responders could turn into responders with increased amount of pulses, and/or more adverse effects as well. The question remains to which extent should TMS pulses be increased, to reach maximum effects. In previous efforts treating depression patients, high frequency stimulation protocol ranging from 10 to 20 Hz for 20 to 30 min (2000–3000 pulses) were commonly adopted over the left dorsal lateral prefrontal cortex, based on the hypothesis that high frequency stimulation could induce “potentiation” like effect on neural transmission and enhance blood flow into the region.

Since 2005, theta burst stimulation (TBS) has been developed as a novel approach in controlling neural activity, with proved efficacy on motor evoked potentials (MEPs) (Huang et al., 2005). The intermittent theta burst stimulation (iTBS) induces potentiation at the given cortical region, while continuous theta burst stimulation (cTBS) suppresses the local brain activity (Martin et al., 2006; Ishikawa et al., 2007). iTBS consists of 3 pulses at 50 Hz and repeated at 5 Hz, 2 s on and 8 s off, leading to 600 pulses at around 3 min. Surprisingly, this short protocol generates similar extent in the excitatory effects on cortex, measured both electrophysiological responses and functional imaging (Huang et al., 2005). Limited studies argued for the use of iTBS in depression treatments (Duprat et al., 2017; Li et al., 2018), including treatment resistant depression.

The questions remain if the short protocol will be as effective when adopted as treatment procedures, or if the procedure is well tolerable in daily application. Blumberger et al. set out to initiate the “THREE-D” trial to compare the effectiveness between iTBS and 10 Hz rTMS in clinical treatments of depression in more than 400 patients (Blumberger et al., 2018). The primary outcome, 17-item Hamilton Rating Scale for Depression, demonstrated clear changes in both groups of patients following 4 weeks of 5 days magnetic stimulation treatment. Notably, the two groups did not show any inter-group differences, suggesting that iTBS is non-inferior to the classical rTMS procedures. While on the other hand, iTBS is much faster and practicable to treat more patients in a day.

In terms of adverse effects, the two groups were similar–more than half subjects reported headache, the most common adverse effects following TMS therapy. The occurrence rates for nausea, dizziness, fatigue, etc. were comparable between the two groups as well. The study also reported more “painful” experience in iTBS group, along with a higher self-reported pain score, but not with more dropout rate in these patients (Blumberger et al., 2018). This suggested that the iTBS is tolerable in daily session treatment and does not accompany more adverse effects. However, in future practices the painful feeling still worth more evaluation for other types of patients.

Cortical plasticity studies demonstrated comparable effects between iTBS and 10 Hz rTMS, even with 600 pulses or 2000–3000 pulses, respectively. Indeed, the treatment effects were similar between the two groups as well. Doubling the dosage of iTBS is not recommended, since this might even lead to inhibitory effects. On the other hand, most previous iTBS studies were performed at the intensity of 80% active motor threshold (AMT), which is around 50–60% of resting motor threshold (RMT). In current study, Blumberger et al. employed 120% of RMT at prefrontal cortex, which could boost the excitatory effects, yet the exact extent still requires further physiological evidences.

In conclusion, the demonstration of new protocol effectiveness, in addition to the classical standard, has largely expanded the possibility to treat more patients per TMS machine per day, and might facilitate the access of patients to treatments. It is also important to consider if the individual variability in treatment effect is due to the different inter-individual plasticity induction responses (Lopez-Alonso et al., 2014). In future, other TBS based treatment protocols would prove their usefulness in clinical treatments for different psychiatric diseases, such as schizophrenia or insomnia (Brunelin et al., 2011; Mensen et al., 2014).

## Author contributions

All authors designed the study together. CH, ZC, and LL wrote the paper together and approved the final version.

### Conflict of interest statement

The authors declare that the research was conducted in the absence of any commercial or financial relationships that could be construed as a potential conflict of interest.
